# Synergistic effect of human uterine cervical mesenchymal stem cell secretome and paclitaxel on triple negative breast cancer

**DOI:** 10.1186/s13287-024-03717-0

**Published:** 2024-04-25

**Authors:** Noemi Eiro, Maria Fraile, Sara Escudero-Cernuda, Juan Sendon-Lago, Luis O. Gonzalez, Maria Luisa Fernandez-Sánchez, Francisco J. Vizoso

**Affiliations:** 1 Research Unit, Hospital de Jove Foundation, Gijón, Spain; 2 Department of Physical and Analytical Chemistry, University of Oviedo, Oviedo, Spain; 3 Experimental Biomedicine Centre (CEBEGA), University of Santiago de Compostela, Santiago de Compostela, Spain

**Keywords:** Secretome, Conditioned medium, Mesenchymal stem cells, hUCESC, Breast cancer, Chemotherapy

## Abstract

**Background:**

Triple-negative breast cancer (TNBC) is the most lethal subtype of breast cancer and, despite its adverse effects, chemotherapy is the standard systemic treatment option for TNBC. Since, it is of utmost importance to consider the combination of different agents to achieve greater efficacy and curability potential, MSC secretome is a possible innovative alternative.

**Methods:**

In the present study, we proposed to investigate the anti-tumor effect of the combination of a chemical agent (paclitaxel) with a complex biological product, secretome derived from human Uterine Cervical Stem cells (CM-hUCESC) in TNBC.

**Results:**

The combination of paclitaxel and CM-hUCESC decreased cell proliferation and invasiveness of tumor cells and induced apoptosis in vitro (MDA-MB-231 and/or primary tumor cells). The anti-tumor effect was confirmed in a mouse tumor xenograft model showing that the combination of both products has a significant effect in reducing tumor growth. Also, pre-conditioning hUCESC with a sub-lethal dose of paclitaxel enhances the effect of its secretome and in combination with paclitaxel reduced significantly tumor growth and even allows to diminish the dose of paclitaxel in vivo. This effect is in part due to the action of extracellular vesicles (EVs) derived from CM-hUCESC and soluble factors, such as TIMP-1 and − 2.

**Conclusions:**

In conclusion, our data demonstrate the synergistic effect of the combination of CM-hUCESC with paclitaxel on TNBC and opens an opportunity to reduce the dose of the chemotherapeutic agents, which may decrease chemotherapy-related toxicity.

**Supplementary Information:**

The online version contains supplementary material available at 10.1186/s13287-024-03717-0.

## Background

The history of breast cancer dates back to 3,000–2,500 BC, the approximate date of the Edwin Smith papyrus [1], the oldest known medical document in the world, which contains the first written description of cancer. Fifteen centuries later (1,500 BC), in the Ebers papyrus, breast cancer with axillary metastasis was described for the first time, evoking the possibility of treatment by surgery, drugs or ignition. Cancer treatment was dominated by surgery and radiotherapy until the mid-1960s [2]. It was at that time, as part of a public programme to identify new anti-tumor compounds in the United States, that samples of Pacific yew (*Taxus brevifolia*) bark were found to exhibit potent cytotoxic activity (1964); paclitaxel was subsequently identified as the active ingredient of the extract [3]. Despite the development of new therapeutic modalities, chemotherapy remains the most widely used antineoplastic therapy in advanced breast cancer due to its high efficacy and despite its adverse effects [4–6].

Among breast cancer molecular subtypes, triple-negative breast cancer (TNBC), defined by the lack of estrogen (ER), progesterone (PR) and human epidermal growth factor receptor 2 (HER-2) expression, is the most lethal subtype of breast cancer with a 5-year mortality of about 40% [7–9]. Currently, chemotherapy is the standard systemic treatment option for TNBC. So, we are treating an old disease using an old therapy. Since, it is of utmost importance to consider the combination of different agents to achieve greater efficacy and curability potential [4]. In this sense, immunotherapy, one of the most significant advances in oncology, introduce biologic agents like monoclonal antibodies (atezolizumab) targeting PD-1 ligand (PD-L1), for example, in TNBC management [10, 11].

A possible innovative alternative could be the use of mesenchymal stem cells (MSC), defined as regenerative undifferentiated cells capable of being differentiated into various cell types [12]. MSC display a wide repertory of paracrine functions due to their secretion of a wide range of soluble factors, such as cytokines or growth factors, and extracellular vesicles. For all of this, MSC have aroused great interest due to their regenerative, anti-inflammatory, immunoregulatory, anti-oxidative stress, anti-fibrotic or anti-microbial properties [13]. Recent studies also show that MSC suppressing tumor growth by inhibiting tumor cell proliferation and inducing apoptosis in cancer cells [14]. Consequently, application strategies based on MSC were recently considered in TNBC [15]. In this regard, we described a new type of mesenchymal stem cell, called human Uterine Cervical Stem Cells (hUCESC), which are obtained from the transitional zone of the cervix of healthy women [16]. The method for obtaining hUCESC (Pap cervical smear or hysterectomy) is much less invasive and painful that those used to obtain other MSC (from the bone marrow or adipose tissue). In addition, hUCESC can be isolated in high quantities, and have a high proliferative rate, making it possible to quickly obtain a huge amount of stem cells or conditioned medium for research and clinical use [16]. Our previous results showed that the secretome or conditioned medium of hUCESC (CM-hUCESC) has a specific anti-tumor effect on proliferation, apoptosis, and invasion of aggressive TNBC cell line MDA-MB-231 and primary mammary carcinoma cultures in vitro [16, 17]. These anti-tumor actions differ from those described for other mesenchymal stem cells [18–20] and may be due, in part, to the fact that CM-hUCESC has higher levels of factors with recognised anti-tumor effects, such as LIGHT (or TNFSF14), Fms-related thyrosine kinase 3 ligand (FLT-3 ligand), interferon gamma-inducible protein-10 (IP-10) and latency-associated protein, compared with CM from adipose-derived mesenchymal stem cells, for example [16].

Based on these data, we have proposed to study the anti-tumor effect of the combination of a chemical agent (paclitaxel) with a complex biological agent (CM-hUCESC) in TNBC.

## Materials and methods

### Primary tumors and breast cancer cell line culture

Primary cell cultures of breast tumors were obtained as previously reported [21, 22]. The estrogen-independent human breast cancer-derived cell line MDA-MB-231 were obtained from the American Type Culture Collection (ATCC, Rockville, MD, USA). MDA-MB-231 cells and primary cells were cultured in DMEM-F12 (Lonza, Visp, Switzerland) supplemented with 10% Fetal Bovine Serum (FBS) (Corning) and 1% penicillin-streptomycin solution (Gibco, Paisley, UK).

### Conditioned medium production

hUCESC were obtained as previously described [16]. Conditioned medium from hUCESC was obtained from 80% cell culture confluence. Afterwards, the cells were washed three times in PBS, and cultured in DMEM-F12 without FBS and antibiotics. After 48 h, the medium was centrifuged for 5 min at 400 g, the supernatant was collected and stored at -80ºC. For the in vivo studies, the CM-hUCESC was lyophilized and then stored at -80 ºC until used and resuspended just before use in the half volume of deionized distilled water (ddH_2_O) to obtain the concentrated CM-hUCESC (2X).

For the production of CM-hUCESC in presence of chemotherapy (CM-hUCESC_chemo_), hUCESC were incubated in presence of a sub-lethal concentration of paclitaxel (10 µM) (Teva Pharma) during 24 h [23]. Afterwards, the cells were washed, and the conditioned medium was produced as previously described.

### Proliferation assay

To determine the effect of the combination of CM-hUCESC or CM- hUCESC_chemo_ with paclitaxel (1 µM, 3 µM and 5 µM) on the proliferation capacity of the aggressive breast cancer cell line MDA-MB-231 and breast cancer primary tumors. Cells were seeded in 96-well plates in DMEM-F12 supplemented with 10% FBS and 1% penicillin-streptomycin. After 24 h, the media was removed and cells were treated with 100 $\mu$L per well of CM-hUCESC or CM-hUCESC_chemo_ with or without paclitaxel, using DMEM-F12 medium as a control, and cultured for 48 h. Finally, 10 $\mu$L of the WST-1 proliferation reagent (Roche) was added and incubated for 4 h at 37 ºC and 5% CO_2_. Proliferation was quantified measuring the absorbance at 450 nm and subtracting the absorbance value at 655 nm.

### Cell cycle and apoptosis assay

Breast cancer cells were seeded in complete medium, washed, and then treated for 24 h in the following conditions: control (DMEM-F12), CM-hUCESC, chemotherapy (paclitaxel 1 µM) and a combination of CM-hUCESC and chemotherapy. Forty-eight hours later, cells were harvested, fixed with ethanol (70%), washed and stained with propidium iodide (50 µg/ml) for 1 h in darkness and cell cycle was analysed by flow cytometry.

Apoptosis analyses were performed using Annexin V-FITC. Briefly, cells were harvested, washed, and resuspended in 1X binding buffer. 5 µl of FITC-Annexin V was added and incubated for 15 min at room temperature in darkness. Finally, 400 µl of 1X binding buffer was added to each tube and analysed.

### Cell invasion assay

Assays were performed in BD BioCoat Matrigel invasion chambers according to the manufacturer’s instructions (BD Biosciences, Madrid, Spain). MDA-MB-231 cells were seeded (50,000 cells) into the upper chamber in DMEM-F12 (control), paclitaxel (1 µM), CM-hUCESC, CM-hUCESC_chemo_ or combination of paclitaxel + CM-hUCESC/CM-hUCESC_chemo_. After incubation for 48 h, cells that had migrated to the lower surface of the filters were fixed in methanol, stained using crystal violet, visualized and counted. Values for cell migration or invasion were expressed as the mean number of cells per microscopic field over four fields per one filter for duplicate experiments.

### Immunoprecipitation (IP) and Western blot (WB)

As described previously, protein A/G PLUS-Agarose (70 µl, sc-2003, Santa Cruz Biotechnology, Dallas, USA) and anti-TIMP-1 antibody (5 µl, sc-365,905, Santa Cruz Biotechnology) or anti-TIMP-2 antibody (5 µl, sc-365,671, Santa Cruz Biotechnology), or IgG1 mouse (5 µl, used as IP control, sc-3877, Santa Cruz Biotechnology) were mixed with 8 ml of CM-hUCESC and incubated overnight at 4 °C in orbital shaking. Then, samples were centrifuged and the supernatant used for functional assays [24].

The precipitate was used to evaluate TIMP-1 and TIMP-2 IP by Western blot. Briefly, after 12% SDS-PAGE electrophoresis, proteins were transferred to a PVDF membrane, blocked, and immunolabeled with TIMP-2 primary antibody (1/200, Thermo-Scientific, Rockford, USA) and second antibody (anti-mouse HRP, 1/3000, Sigma). Signal was detected with the SuperSignal West Pico Plus (Thermo Scientific, Rockford, USA), and visualized by placing the blot in contact with standard X-ray film.

### Isolation and characterization of CM-hUCESC_chemo_ extracellular vesicles

Isolation of extracellular vesicles (EVs)was performed by differential ultracentrifugation, as described previously [25]. The pellet obtained by ultracentrifugation at 100,000 g for 70 min corresponding to the fraction containing the EVs was resuspended in 0.1 μm filtered PBS with sucrose at 1% (Sigma-Aldrich). The presence of EVs in the sample was detected by Transmission Electron Microscopy (TEM) JEM-1011 (JEOL, Japan) at 100 kV with a previous fixing with 2% paraformaldehyde and dyed with 2% phosphotungstic acid. The EVs and Taxol-encapsulated EVs were characterized by a Pierce™ bicinchoninic acid assay kit (Thermo Fisher Scientific, USA) for total protein quantification. The size distribution and particle concentration were determined by Nanoparticle Track Analysis (NTA) Nanosight LM10 (Malvern Panalytical, United Kingdom).

EVs were characterized by flow cytometry using specific antibodies: anti-CD9 antibody (FITC) (Abcam, United Kingdom), anti-CD63 (APC) (Bio-Rad, USA) and anti-CD81 antibody (PE) (Bio-Rad, USA). Also, the Molecular Probes™ CellTrace™ Calcein Violet, AM (Invitrogen, USA) was used to ensure EVs integrity. The fluorescence was monitored by a Cytoflex S Flow Cytometer using a Violet Laser (405 nm) and its light Side Scattering (SSCviolet).

### Determination of paclitaxel by HPLC-ESI-Q-TOF

For paclitaxel determination, EVs, CM-hUCESC_chemo_ and hUCESC cells were lysed as previously described [23]. The lysate were dried by speed vacuum (Concentrator 5301 Eppendorf), resuspended in the chromatographic initial phase and spiked with Docetaxel at 200 ng/mL as Internal Standard and injected into the HPLC (Dionex Ultimate 3000). The chromatographic separation was performed with an InfinityLab Poroshell 120 EC-C18 column using ultrapure water (LabWater, Purelab Flex system ELGA, UK) as phase A and acetonitrile (Fischer Scientific, USA) as phase B, both with formic acid at 0.1% (Acros Organics, Germany). The chromatographic gradient was performed at 0.2 mL/min (0 min–40% B, 2 min–40% B, 10 min–60% B). Paclitaxel was detected by an ESI-QTOF spectrometer (Bruker Impact II model) with the following settings: capillary voltage 3500 V, dry gas at 6 L/min, dry temperature of 250ºC, mass range (m/z) between 50 and 1500 and spectra rate of 1.00 Hz.

### Animal studies

Six weeks old female NMRI Fox1 nu/nu mice were used for xenografting studies. Twenty-eight mice (7 per group) were injected into the mammary fat pads with 2.5 × 10^6^ MDA-MB-231-luc- cells in matrigel. Ten days after cell injection, the mice were treated until day thirty: group 1 (control): DMEM-F12 culture medium + saline solution; group 2: CM-hUCESC (2X) intraperitoneally (200 µl) 3 times/week; group 3: paclitaxel 10 mg/kg intraperitoneally once/week and group 4: CM-hUCESC (2X) intraperitoneally (3 times/week) + paclitaxel 10 mg/kg intraperitoneally (once per week). Tumor growth was monitored by luminescence, after luciferin injection (150 mg/kg), mice were anesthetized using 2.5% isoflurane and imaged using the In Vivo Imaging System (IVIS, Caliper Life Sciences, Alameda, CA, USA) at day 0, 7, 14, 21 and 30. An intensity map was obtained using the Living Image software (Caliper Life Sciences). The software uses a color-based scale to represent the intensity of each pixel (from blue representing low to red representing high).

To investigate the effect of CM- hUCESC_chemo_, a similar study was conducted as follows: group 1 (control) (*n* = 4): DMEM-F12 culture medium + saline solution; group 2 (*n* = 7): CM-hUCESC_chemo_ intraperitoneally (200 µl) 3 times/week; group 3 (*n* = 7): paclitaxel 5 mg/kg intraperitoneally once/week and group 4 (*n* = 7): CM-hUCESC_chemo_ intraperitoneally (3 times/week) + paclitaxel 5 mg/kg intraperitoneally (once per week).

All animals were euthanized by carbon dioxide inhalation. The manuscript reporting adheres to the ARRIVE (Animal Research: Reporting of In Vivo Experiments) 2.0 guidelines.

### Statistical analysis

Data analysis and statistics (t-tests, ANOVA, Kruskal-Wallis) were conducted with PASW Statistics 18 (San Diego, CA, USA) and a p value < 0.05 was considered statistically significant. We have also calculated the adjusted p-values using the Benjamini-Hochberg procedure, also known as the False Discovery Rate (FDR) procedure, used to control the expected proportion of false discoveries.

## Results

### Synergistic effect of CM-hUCESC in combination with paclitaxel on breast cancer cell proliferation

**Fig. 1:**
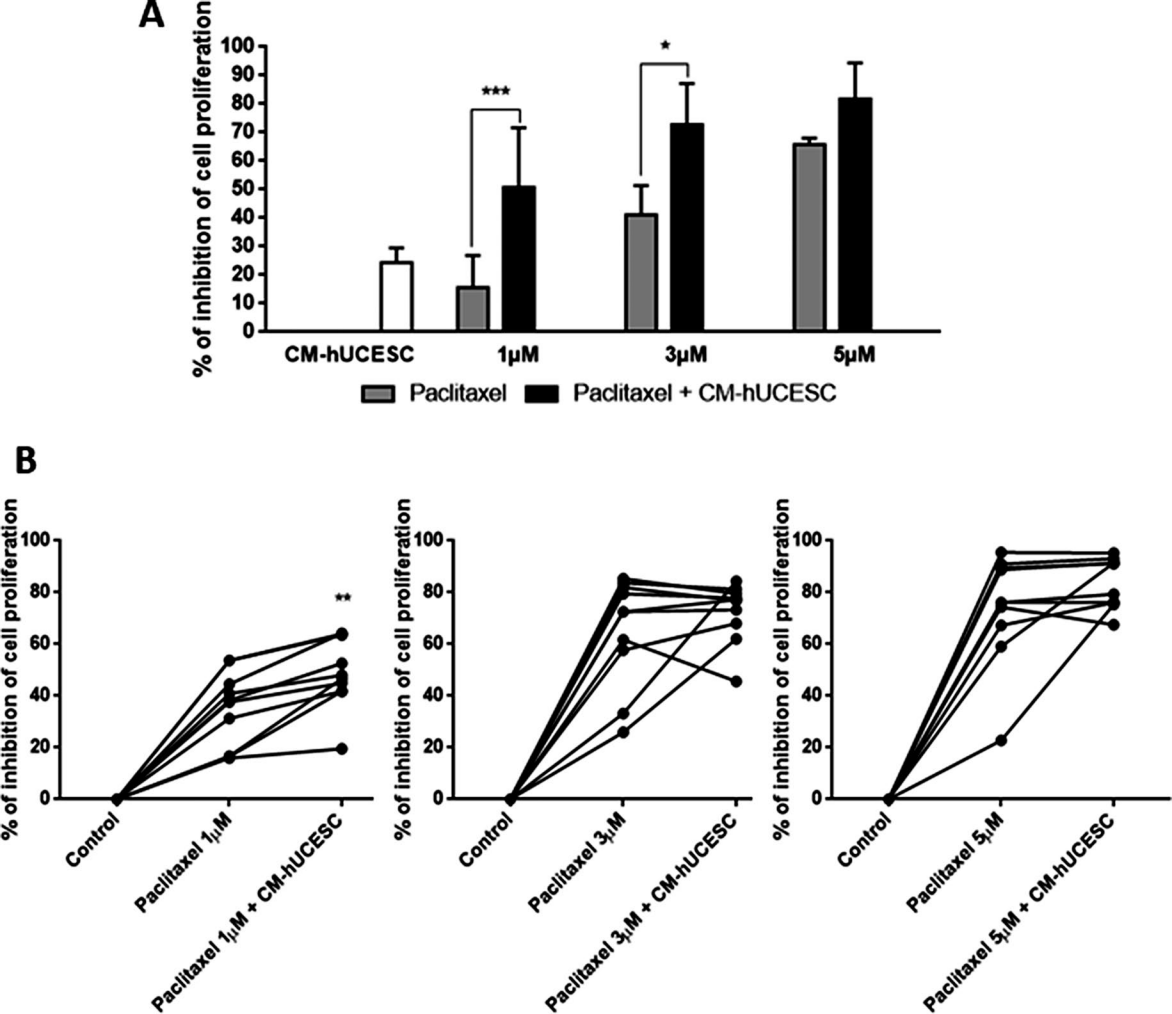
**(A)** Relative proliferative capacity of MDA-MB-231 cells treated for 48 h with CM-hUCESC, paclitaxel (1 µM, 3 µM and 5 µM) and the combination of paclitaxel (1 µM, 3 µM and 5 µM) + CM-hUCESC. **(B)** Relative proliferative capacity of primary tumor cells from TNBC treated for 48 h with CM-hUCESC, paclitaxel (1 µM, 3 µM and 5 µM) and the combination of paclitaxel (1 µM, 3 µM and 5 µM) + CM-hUCESC. *p < 0.05, ***p < 0.0001

As shown in Fig. 1, MDA-MB-231 cells and primary breast cancer cells treated with CM-hUCESC and/or paclitaxel showed a significant inhibition of proliferation compared with the control (DMEM-F12). As expected, the higher the concentration of paclitaxel showed the greater inhibition of cell proliferation among paclitaxel treated cells. However, the combination of paclitaxel 1 µM with CM-hUCESC significantly increased the inhibition of proliferation of MDA-MB-231 cells and primary tumors, showing a synergic effect (*p* < 0.0001, for both, adjusted p value = 0.0007, for both). In fact, the addition of CM-hUCESC to a low dose of paclitaxel showed similar or even superior proliferation inhibition to that exhibited with a higher concentration of paclitaxel; in this sense, cells treated with 1 µM paclitaxel + CM-hUCESC showed a greater inhibition of cell proliferation to that shown by cells treated with 3 µM paclitaxel + CM-hUCESC.

### Effect of combination of CM-hUCESC and paclitaxel on cell cycle and apoptosis

**Fig. 2:**
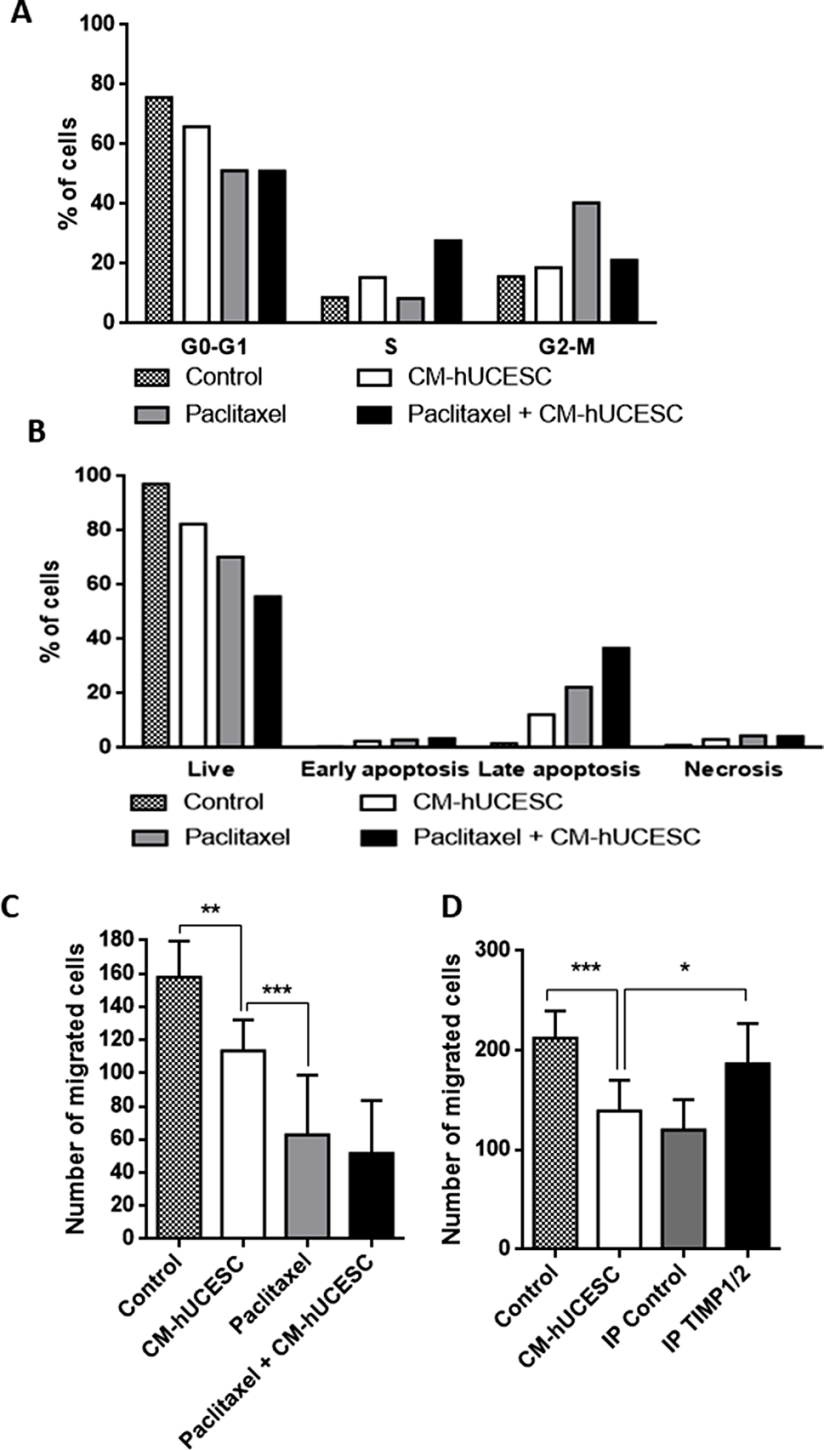
**(A)** Cell cycle analysis of MDA-MB-231 cells treated for 48 h with control (DMEM-F12 without FBS), CM-hUCESC, paclitaxel (1 µM) or the combination of paclitaxel (1 µM) + CM-hUCESC, and then subjected to flow cytometry using propidium iodide (PI). Percentage of cells (mean + standard deviation) in each phase is shown. **(B)** Apoptosis was determined in MDA-MB-231 cells cultured for 48 h with control (DMEM-F12 without FBS), CM-hUCESC, paclitaxel (1 µM) or the combination of paclitaxel (1 µM) + CM-hUCESC, by flow cytometry using Annexin V/PI. Annexin V+/PI- and Annexin V+/PI + indicated early and late apoptosis, respectively. **(C)** Invasive capacity of MDA-MB-231 cells treated for 48 h with control (DMEM-F12 without FBS), CM-hUCESC, paclitaxel (1 µM) or the combination of paclitaxel (1 µM) + CM-hUCESC in Matrigel invasion chambers. **(D)** Invasive capacity of MDA-MB-231 cells treated for 48 h with control (DMEM-F12 without FBS), CM-hUCESC, IgG IP and CM-hUCESC lacking TIMP-1 and − 2 (IP TIMP-1/2) in Matrigel invasion chambers. Data represent the mean ± SD. *p < 0.05; ***p < 0.0001

Given that paclitaxel + CM-hUCESC significantly decreased proliferation of MDA-MB-231 cells, we next evaluated cell cycle and apoptosis as possible mediators. As shown in Fig. 2, cells treated with paclitaxel, CM-hUCESC or the combination paclitaxel + CM-hUCESC decreased their G0-G1 phase in relation to cells treated with control (DMEM-F12) but increased their G2-M phase in relation to control (Fig. 2A).

Treatment of MDA-MB-231 cells with paclitaxel + CM-hUCESC induced a decrease of live cells (Annexin-/PI-) compared with control, CM-hUCESC alone or paclitaxel alone and an increase of Annexin V+ / PI + cells suggesting that the combination of both treatments induces late apoptosis (Fig. 2B).

### Inhibition of breast cancer cell invasiveness by combining CM-hUCESC with paclitaxel

In order to evaluate the impact of the combination of both treatments on cell invasion capacity, we have carried out a functional study treating the breast cancer cell line MDA-MB-231 with DMEM-F12 (control), CM-hUCESC, paclitaxel (1 µM) and the combination of paclitaxel + CM-hUCESC. As shown in Fig. 2C, CM-hUCESC significantly inhibited MDA-MB-231 cell invasion capacity (28%, *p* < 0.0001 compared with control (adjusted p value = 0.004)) but the inhibition of the invasion capacity is higher by paclitaxel (61%) (*p* < 0.0001 compared with CM-hUCESC, (adjusted p value = 0.004)). Although, the combination of both (paclitaxel + CM-hUCESC) showed the highest inhibition of breast cancer cell invasiveness there was no significant difference compared with paclitaxel alone.

### TIMP-1 and TIMP-2 mediate the anti-tumoral effect induced by CM-hUCESC

We have previously described that some factors secreted by mesenchymal stem cells could be responsible for their therapeutic effects [26] and that the tissue inhibitors of metalloproteinases TIMP-1 and − 2 are present at high levels in CM-hUCESC [24]. To establish if TIMP-1 and − 2 are involved in the anti-tumoral effect induced by CM-hUCESC, they were immunoprecipitated (IP) from the CM-hUCESC with specific antibodies. IgG IP was used as control. A Western blot was carried out demonstrate the immunoprecipitation of TIMP-2 (supplementary Fig. 1). In the case of TIMP-1 the molecular weight of the light chain of the anti-TIMP-1 antibody (25 kDa) used in the immunoprecipitation is close to the molecular weight of the TIMP-1 protein (29 kDa). Due to the excess of the antibody use during the immunoprecipitation (to ensure that all TIMP-1 is immunoprecipitated) and the intensity of the band, this band masks the detection of any protein of interest with a molecular weight close to 25 kDa (data not shown). Then, a cell invasion assay was carried out with MDA-MB-231 cells treated with whole CM-hUCESC or without TIMP-1 and − 2. Complete CM-hUCESC significantly decreased migration of cancer cell line as compared with control (*p* < 0.0001, adjusted p value = 0.0007) and with CM-hUCESC lacking TIMP-1 and − 2 (IP TIMP-1/2) (*p* = 0.004, adjusted p value = 0.008) (Fig. 2D). Moreover, invasion of confluent MDA-MB-231 cells induced by CM-hUCESC lacking TIMP-1 and − 2 was similar to that induced by control.

### In vivo effect of combination of CM-hUCESC and paclitaxel

**Fig. 3:**
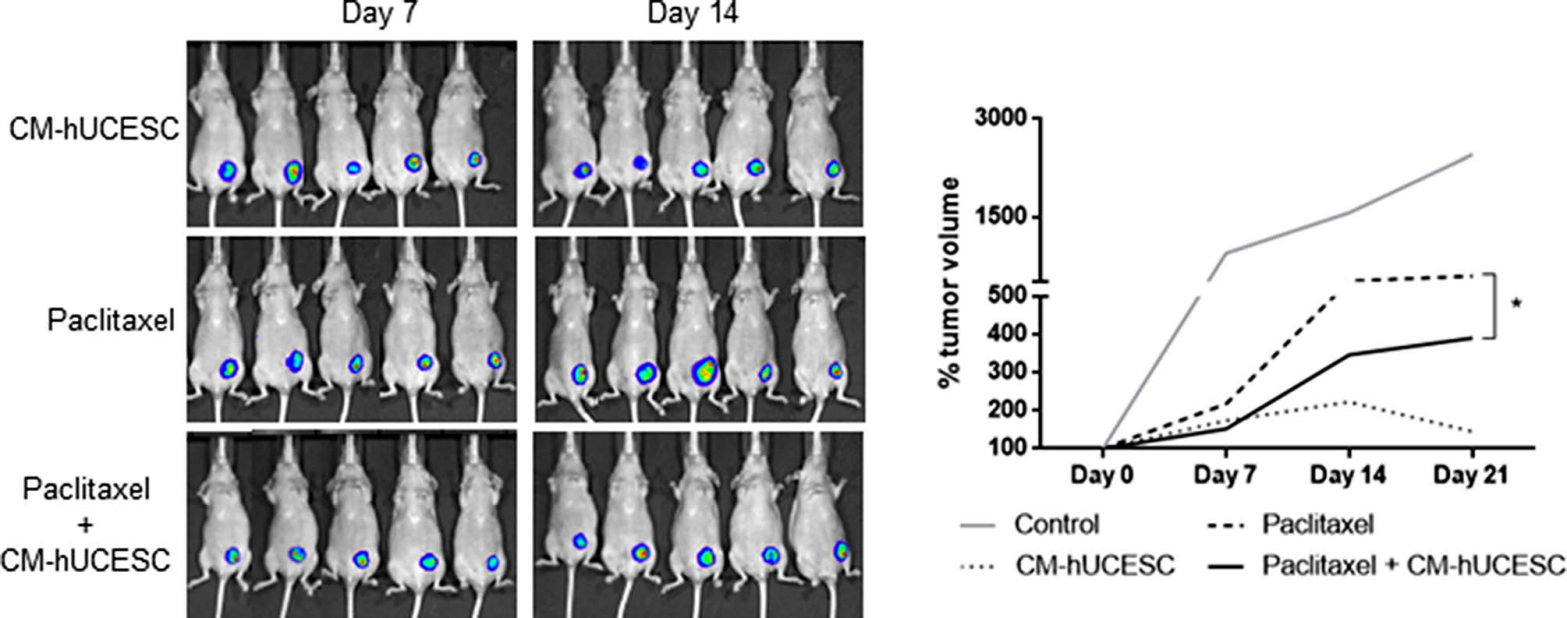
Representative images from mice treated with CM-hUCESC, paclitaxel 10 mg/kg and the combination of paclitaxel 10 mg/kg + CM-hUCESC taken at 7 and 14 days and tumor volume which was determined by measuring luminescence since day 0 until day 21

We have evaluated the effect of intraperitoneal administration of paclitaxel (10 mg/kg) and CM-hUCESC in vivo using NMRI fox1 nu/nu mouse tumor xenograft model. Mice were injected with MDA-MB-231 cells stably transfected with the luciferase vector in the mammary fat pad. When the tumor became visible, mice were injected intraperitoneally, as described above, with DMEM-F12 (control), CM-hUCESC, paclitaxel or the combination of paclitaxel + CM-hUCESC. The evaluation of tumor growth could be analysed until day 21 because at day 28/30 many of the tumors showed necrosis. As it can be observed in Fig. 3, mice treated with paclitaxel showed a significantly reduction of the tumor growth compared with control (*p* < 0.0001; adjusted p value = 0.0007) and it was confirmed that the combination of chemotherapy treatment (paclitaxel) with the biological product (CM-hUCESC) significantly reduces tumor growth, especially at day 14 (*p* = 0.025; adjusted p value = 0.036). Although mice treated with CM-hUCESC showed less tumor growth than mice treated with paclitaxel + CM-hUCESC, there was no significant difference compared to the combination of treatments.

### Effect of CM-hUCESC_chemo_ and its combination with paclitaxel

**Fig. 4:**
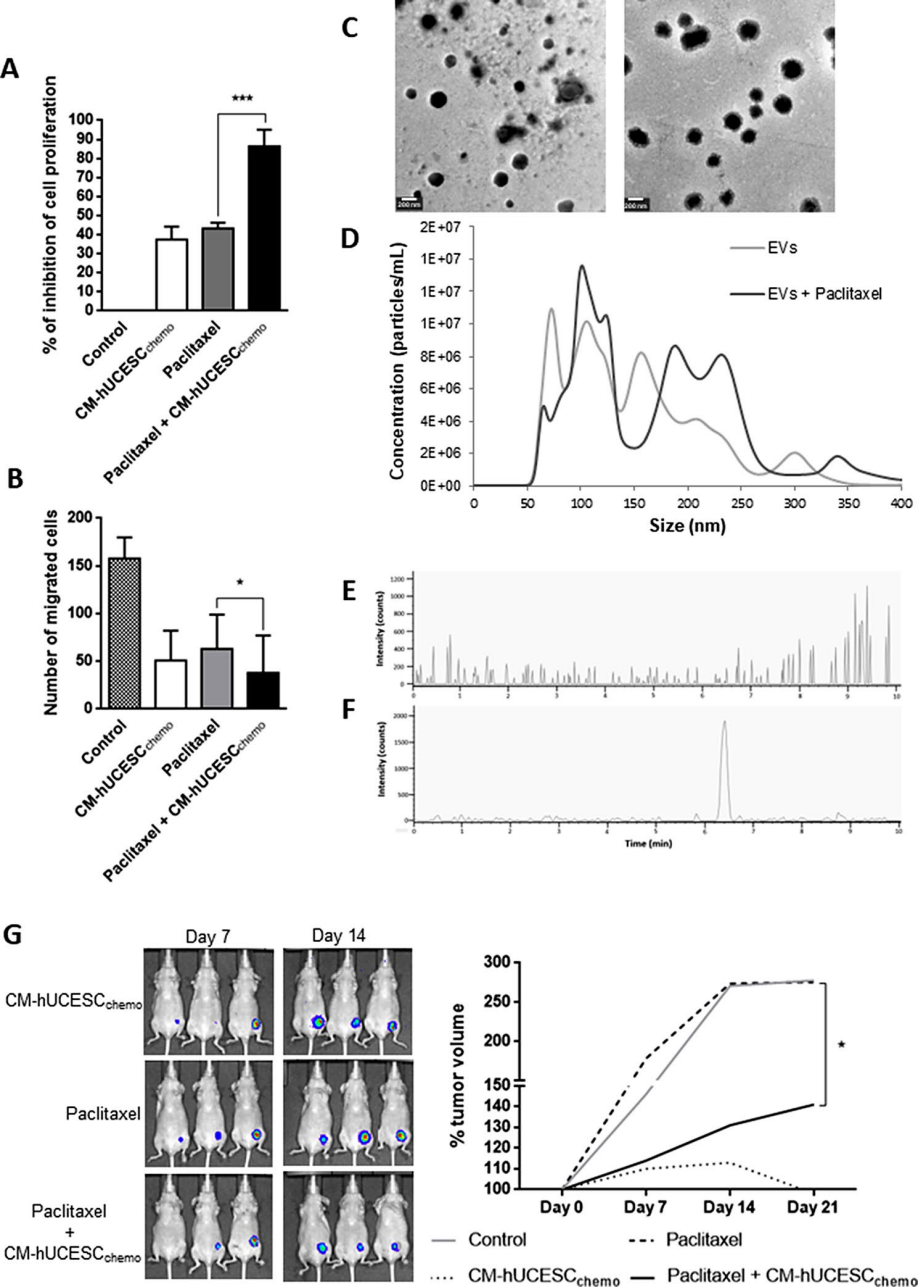
**A)** Relative proliferative capacity of MDA-MB-231 cells treated with control (DMEM-F12 without FBS), CM-hUCESC_chemo_ (pre-conditioning of hUCESC with paclitaxel previously of CM production), paclitaxel (1 µM) and the combination of paclitaxel (1 µM) + CM-hUCESC_chemo_. **B)** Invasive capacity of MDA-MB-231 cells treated with control (DMEM-F12 without FBS), CM-hUCESC_chemo_, paclitaxel (1 µM) and the combination of paclitaxel (1 µM) + CM-hUCESC_chemo_. **C**) Transmission Electron Microscopy micrographs for control EVs (left) and paclitaxel-loaded EVs (right). **(D)** Size Distribution graphs by NTA for control EVs and drug loaded EVs. **(E)** Extracted Ion Chromatogram by HPLC-ESI-TOF at paclitaxel mass/charge for control EVs. **(F)** Extracted Ion Chromatogram by HPLC-ESI-TOF at paclitaxel mass/charge for drug-loaded EVs. **(G)** Representative images from mice treated with CM- hUCESC_chemo_, paclitaxel 5 mg/kg and the combination of paclitaxel 5 mg/kg + CM-hUCESC taken at 7 and 14 days and tumor volume which was determined by measuring luminescence since day 0 until day 21

In order to improve the potential of CM-hUCESC and the effect of its combination with paclitaxel, hUCESC were cultured with a sublethal dose of chemotherapeutic prior to CM-hUCESC production. Subsequently, we evaluated the effect of CM-hUCESC_chemo_ and its combination on the proliferative and invasive capacity of MDA-MB-231, showing that it significantly decreased their functional capacities (Fig. 4A and B). CM-hUCESC_chemo_ decreased the proliferation of tumor cells (37.4%) similarly to paclitaxel (43.2%), but their combination showed a significant inhibition of proliferation by up to 86.4% (*p* < 0.0001; adjusted p value = 0.0007). Regarding the regulation of tumor cell invasiveness, the combination of paclitaxel with CM-hUCESC_chemo_ significantly decreased the capacity of cell invasion of tumor cells (compared with paclitaxel alone (*p* = 0.045; adjusted p value = 0.052)).

### Characterization of CM-hUCESC_chemo_ extracellular vesicles

EVs and paclitaxel-loaded EVs were characterized by the following techniques. As can be seen in Fig. 4C, the presence of rounder vesicles in both samples was detected by Transmission Electron Microscopy (TEM). The paclitaxel-loaded EVs were slightly larger than the control EVs and the first ones possessed what it seemed like a lower electron density corona around them. For a better knowledge of the size distribution of the samples, Nanoparticle Tracking Analysis (NTA) analyses were performed. The size distribution graphs (Fig. 4D) also show an increase in size for paclitaxel-loaded EVs with a diameter media of 190 ± 8 nm and a mode of 132 ± 28 while control EVs have a diameter media of 130 ± 6 and a mode of 97 ± 14 nm. This is in agreement to previous studies: the EVs size increased proportionally to the amount of entrapped drug [27, 28]. However, despite the differences in size, the control EVs and the paclitaxel-loaded EVs show the same markers (CD9, CD63 and CD81) (Supplementary Fig. 2). Regarding particle concentration calculated by NTA, an increase in particle production was observed for paclitaxel-treated cells (1.34 + E11 ± 0.21 + E11 particles/mL vs. 0.47 + E11 ± 0.03 + E11 particles/mL for non-loaded EVs). This increase in particle production could be due to cellular stress produced by the paclitaxel treatment [23]. The bicinchoninic acid assay (BCA) also showed that this increase in particle concentration is correlated with a total protein rise (17.2 ± 1.6 vs. 14.4 ± 1.6 µg/mL for paclitaxel-loaded EVs and control EVs, respectively).

### Determination of paclitaxel in CM-hUCESC_chemo_

Paclitaxel determination was performed in EVs and CM-hUCESC_chemo_ samples and hUCESC by HPLC-ESI-QTOF. Figure 4E and F show the Extracted Ion Chromatogram of paclitaxel (m/z = 854.34 ± 0.01) for EV samples. As can be seen, paclitaxel was not detected in the control sample (Fig. 4E), but its presence is confirmed in the drug loaded vesicles (Fig. 4F).

### Reducing tumor growth by combining CM-hUCESC_chemo_ with a lower dose of paclitaxel

Mice were injected intraperitoneally with DMEM-F12 (control), CM-hUCESC_chemo_, paclitaxel or the combination of paclitaxel + CM-hUCESC_chemo_. The evaluation of tumor growth could be analysed until day 21 because at day 28/30 many of the tumors showed necrosis. As it can be observed in Fig. 4G, mice treated with a low dose of paclitaxel (5 mg/kg) showed a similar tumor growth than control mice. Mice treated with CM-hUCESC_chemo_ showed a significant reduction of tumor growth, by almost of 60%, compared with paclitaxel alone (113% vs. 274% tumor volume at day 14, respectively, *p* = 0.032; adjusted p value = 0.042), and by 20% compared with the combination of both treatment (113% vs. 131% tumor volume at day 14, respectively, no significant difference). However, the combination of paclitaxel (5 mg/kg) + CM-hUCESC_chemo_ showed a significant reduction, by almost of 50%, of tumor growth compared with paclitaxel alone (131% vs. 274% at day 14, respectively, *p* = 0.016; adjusted p value = 0.029), which means that a low dose of paclitaxel combined with CM-hUCESC can inhibit TNBC tumor growth in vivo.

## Discussion

The results of the present study demonstrate the synergistic effect of the combination of CM-hUCESC with paclitaxel. First, the effect was evidenced in vitro through decreased cell proliferation and invasiveness of tumor cells and induction of apoptosis. Second, the effect was confirmed in a mouse tumor xenograft model showing that the combination of the chemotherapeutic agent with the secretome of hUCESC has a significant effect in reducing tumor growth. Third, pre-conditioning hUCESC with a sub-lethal dose of paclitaxel enhances the effect of its secretome and in combination with paclitaxel, even allows to reduce the dose of paclitaxel in vivo.

Controversial reports have been published regarding the pro- or anti-tumor effect of MSC [14, 29, 30], largely justified by the tissue of origin of MSC, the variability of stem cell donors and experimental conditions, among others [31, 32]. The source of MSC and the type of tumor seem to be the most influential factors in this controversy. In fact, many studies are contradictory regarding the effect on breast cancer cells of bone marrow (BM)-MSC and adipose tissue-derived (AD)-MSC or their derived-products [14]. However, relevant and consistent results are found regarding the effect of MSC of uterine (i.e., cervix, endometrium) or reproductive tissue origin (i.e., umbilical cord) on breast and ovarian cancer cells, as shown by hUCESC previously [16, 33], as well as in the present study through the effects on proliferation, apoptosis, and tumor-cell invasiveness. This leads to consider the importance of the tissue origin of MSC as it may influence their ability to regulate homeostasis in tissues highly exposed to external aggressions such as the uterine cervix.

The MSC-derived secretome is composed of cytokines, growth factors and extracellular vesicles, among others, which represent a therapeutic alternative that avoids the drawbacks and offers solutions to the limitations associated with cell therapy, such as: (i) avoids transplantation of live and proliferative cells; (ii) offers similar safety assessment, dosage and potency to conventional pharmaceutical agents; (iii) offers easy storage; (iv) offers a more practical and cheaper clinical application; (v) allows the biological product obtained for therapeutic applications to be modified to suit the patient’s needs, among others [26]. Other drawbacks of MSC cell therapy with regard to TNBC treatment have been reported, such as the immune rejection of the transplant due to repeated injections or prolonged exposure to MSC, leading to a poor MSC survival and function at the tumor site [15]. Although many research studies and clinical trials continue to approach cell therapy as an MSC-based therapy, more and more studies are being conducted based on the use of the secretome or cell-derived products [16, 33–37], as it is known that the mechanism of action of MSC is primarily paracrine.

Our results have shown that the secretome of hUCESC alone inhibits cell proliferation, induces apoptosis, regulates the cell cycle, and reduces the invasiveness of aggressive breast cancer cells, which differs from the results of the secretome of other types of MSC such as AD-MSC [38], but also potentiates these same effects of chemotherapy, unlike secretome of umbilical cord (UC)-MSC which did not significantly affect its cytotoxic, anti-migratory or anti-invasive effects on tumor cells [39]. Thus, the synergistic effect evidenced led us to investigate the impact of priming hUCESC with paclitaxel, before CM production, and its combination with chemotherapy on tumor cell behaviour. The use of a sublethal dose [23, 40] has allowed to potentiate the effect of paclitaxel in vitro and in vivo, but also to reduce the dose of paclitaxel administered to the mice, with a possible reduction of the side effects associated with the use of chemotherapy.

The observed effects could be partly due to the incorporation of paclitaxel into the extracellular vesicles released into the environment by the hUCESC, since the presence of paclitaxel in the EVs present in the CM-hUCESC_chemo_ has been observed. The incorporation of paclitaxel into EVs produced an increase in particle size and number, probably due to cellular stress induced by the paclitaxel treatment. Most of EVs present in this CM-hUCESC can be classified into exosomes by size (30–150 nm). These EVs are of great interest as a possible alternative to exploiting the properties of MSC, or at least part of them, due to their advantages such as better safety profile, lower immunogenicity, the ability to cross biological barriers (gastrointestinal barrier and blood–brain barrier), and to avoid immune rejection [14, 41–43]. EVs are one of the interaction pathways between MSC and breast cancer tumor cells, MSC-derived EVs (MSC-EVs) can transport molecules, such as proteins and nucleic acids, through which they exert inhibitory or promoter effects on breast cancer cells [13, 44]. At the same time, MSC-EVs provide new therapeutic options such as being carriers for drug delivery [45]. Indeed, other studies demonstrated the therapeutic efficacy of either paclitaxel-loaded MSC-CM [46] or -EVs [47, 48] and even that paclitaxel incorporated into MSC-EVs induced the same effects at a reduced concentration as the direct use of the chemotherapeutic agent, indicating that chemotherapeutic-loaded MSC-Es have a specific and more efficient property to attack tumors [23], due to the internalisation of EVs by target cells. The loading process can be enhanced by electroporation, lipofection, sonication or extrusion [49], instead of use a passive incorporation, as genuinely appear to display the hUCESC. Therefore, our study is in the current research line that point to the special interest of CM and EVs derived from MSC for the treatment of TNBC [48, 50–52].

There are other mechanisms that could explain the anti-tumor effect induced by CM-hUCESC. Thus, for example, the involvement of matrix metalloproteinases (MMPs) in cancer progression through the processes of cell migration and invasion has been widely reported [53–57]. The activity of MMPs is controlled by tissue inhibitors of metalloproteinases (TIMPs) to prevent excessive proteolysis, tissue damage, migration and invasion of tumor cells through basement membrane degradation. Of fact, it has been reported that the anti-tumor effect of MSC may be partly related to the activity of the TIMP-1 and TIMP-2 [58]. A previous proteomic study has revealed the presence of high levels of TIMP-1 and TIMP-2 in CM-hUCESC [59], and in the present study we have evidenced that these factor secreted by hUCESC contribute to the regulation of tumor aggressiveness through the inhibition of tumor cell invasion, as well as being implicated in other non-tumor processes induced by CM-hUCESC [24].

In summary, this study on CM-hUCESC provided several novelties, such as (i) the combination of the biological product CM-hUCESC with chemotherapy (Paclitaxel); (ii) the demonstration of the effect of this combination, in vitro, on cell proliferation, invasiveness and apoptosis of breast cancer tumor cells (MDA-MB-231 and/or primary tumor cells); (iii) the anti-tumor effect was confirmed in a mouse tumor xenograft model showing that the combination of both products has a significant effect in reducing tumor growth; (iv) pre-conditioning hUCESC with a sub-lethal dose of paclitaxel enhances the effect of its secretome, in vitro and in vivo; (v) the combination of the secretome from pre-conditioned hUCESC with paclitaxel reduced significantly tumor growth and even allows to diminish the dose of paclitaxel, in vivo; (vi) mechanism of action based on soluble factors present in the secretome, such as TIMP-1 and TIMP-2, and extracellular vesicles (EVs).

## Conclusions

In conclusion, our data indicate that CM-hUCESC enhances the chemotherapy effects on triple negative breast cancer cells and opens an opportunity to reduce the dose of the chemotherapeutic agents, which may decrease chemotherapy-related toxicity. This effect is in part due to the action of their EVs and soluble factors, such as TIMP-1 and − 2. In addition, the possibility to obtain EVs loaded with chemotherapeutic agents open the scenario for more selective anti-tumor strategies. We consider that our study also suggests the interest of future studies on the mechanisms underlying, such as PI3K/AKT/NFκB pathway and on the trophism of the MSC derived-EVs toward tumors, as well as to explore the possible impact of CM-hUCESC to relieve chemotherapy-induced tissue injuries, such as cardiotoxicity, nephrotoxicity, pulmonary toxicity, and reproductive tract toxicity.

### Electronic supplementary material

Below is the link to the electronic supplementary material.

Supplementary Material 1

Supplementary Material 2

## Data Availability

The data presented in this study are available on request from the corresponding author.

## Declarations

The animal studies were conducted according to the ARRIVE guidelines (Animal Research: Reporting of In Vivo Experiments) and approved by the Universidad de Santiago de Compostela Ethics Committee for Animal Experiments (Project title: Breast cancer: tumor cell and stroma as therapeutic targets of stem cells and vitamin D analogues. Approval no: 15010/16/001, March 2, 2016).

## Abbreviations

(AD)-MSC: adipose tissue-derived mesenchymal stem cells
(BM)-MSC: bone marrow mesenchymal stem cells
CM-hUCESC: secretome derived from human Uterine Cervical Stem cells
ER: estrogen receptor
EVs: extracellular vesicles
HER-2: human epidermal growth factor receptor 2
hUCESC: human Uterine Cervical Stem Cells
MMPs: matrix metalloproteinases
MSC: mesenchymal stem cells
PD-L1: PD-L1
PR: progesterone
TEM: Transmission Electron Microscopy
TIMPs: tissue inhibitors of metalloproteinases
TNBC: triple-negative breast cancer
