# Supplementary figures and images for: Synergistic effect of human uterine cervical mesenchymal stem cell secretome and paclitaxel on triple negative breast cancer
Source: Stem Cell Res Ther. 2024 Apr 25;15:121. doi: 10.1186/s13287-024-03717-0 (PMC11044487; doi:10.1186/s13287-024-03717-0)

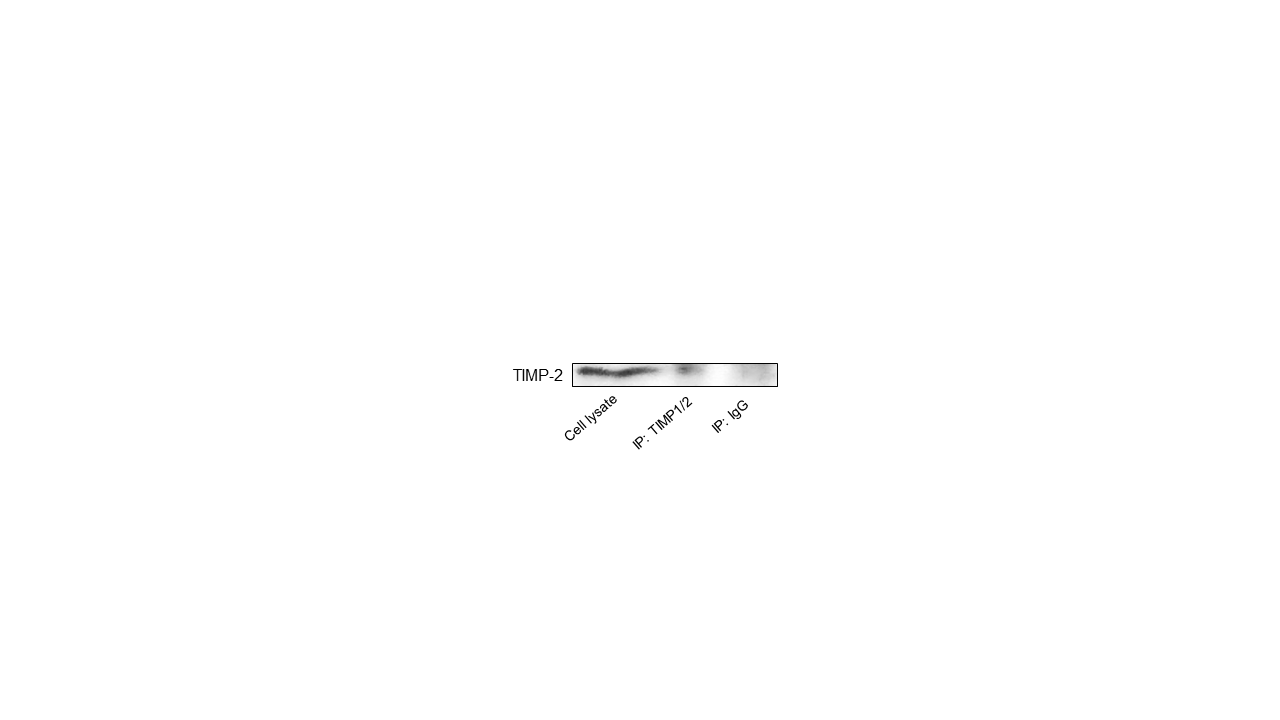

Supplement: Supplementary file 1 — Supplementary Material 1 [file 13287_2024_3717_MOESM1_ESM.tif]

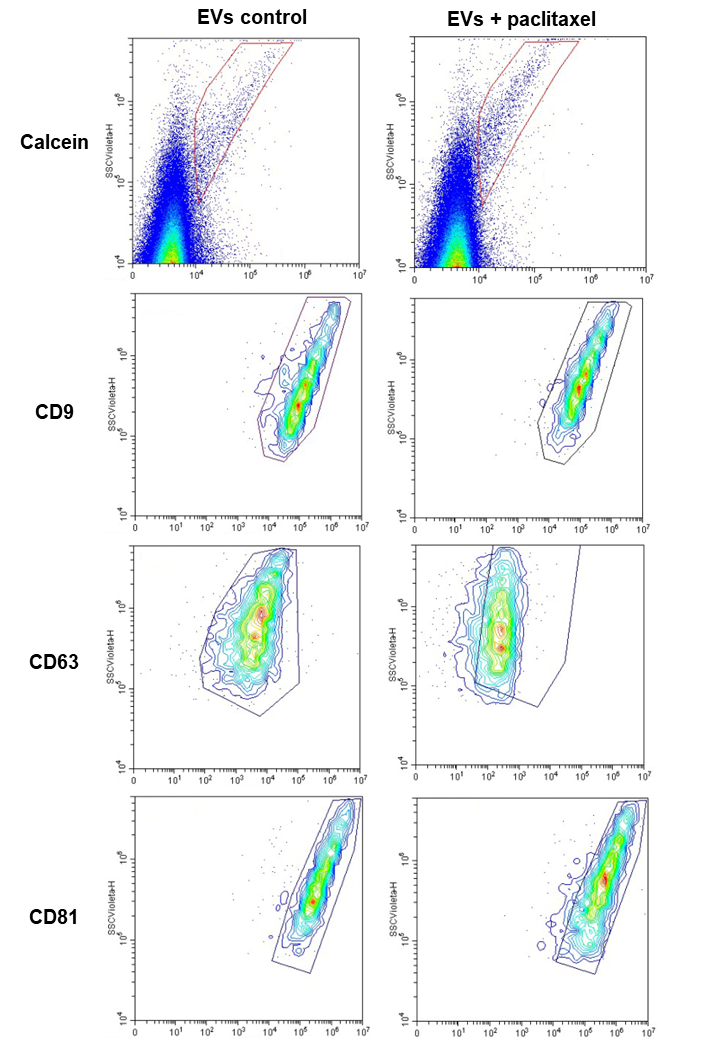

Supplement: Supplementary file 2 — Supplementary Material 2 [file 13287_2024_3717_MOESM2_ESM.tif]
